# How to increase children and young adults' understanding and awareness of agricultural careers: the extent of stakeholder consensus, constructiveness and criticism in written submissions to a UK government inquiry

**DOI:** 10.3389/fsoc.2026.1761629

**Published:** 2026-02-06

**Authors:** Claire Toogood

**Affiliations:** 1 Harper Adams Business School, Harper Adams University, Shropshire, United Kingdom; 2 School of Business and Law, Birmingham Newman University, Birmingham, United Kingdom; 3 Graduate Futures Institute, Sheffield, United Kingdom

**Keywords:** agriculture, careers, qualitative content analysis, SDG 8, children, young adults

## Abstract

This research aimed to establish whether there is consensus amongst stakeholders on how to increase agricultural understanding and awareness amongst children and young people, including consideration of stakeholder constructiveness and criticism, in order to contribute to broader understanding of why significant challenge persists in maintaining and growing a sustainable agricultural workforce in the UK. Qualitative content analysis (QCA) was used to review 25 submissions to a recent UK government inquiry on this topic. Responses were included in the analysis if they had answered the inquiry question focused on improving understanding and awareness of career opportunities in land-based sectors amongst children and young adults, and if their question response or their organisational/individual description included any of the following keywords: farm, farmer, farming, agriculture, agricultural, or agri. A high degree of consensus amongst stakeholders was observed, and responses were typically more constructive than critical, with a clear willingness to take action to tackle sector challenges. Specific themes and ideas around the need for high quality career and labour market information and support, engagement with education, collaboration, partnership and policy, and public presence and awareness were identified. There was considerable alignment between existing research, career theory, and the inquiry submissions. It is recommended that agricultural stakeholders are empowered to develop further collaborations, with appropriate government policy and financial support in place, to help them enact their shared vision of constructive change.

## 1 Introduction

As explored in Social Cognitive Career Theory (SCCT), career development, awareness and understanding is shaped by contextual factors (Lent et al., 1994). Each individual's socio-cultural environment, support and influences affect their career development (Howard and Ferrari, 2022; Sullivan and Baruch, 2009); this evolving and iterative process begins in childhood and continues throughout the individual's whole life with levels of vocational maturity typically increasing with age (Hartung et al., 2005; Watson and McMahon, 2005). However, during this personal development process, young people may not always have realistic expectations of their potential career and its trajectory, their job aspirations and real-world job availability are demonstrably mismatched in worldwide studies (Hoff et al., 2022; OECD, 2025).

Understanding how young people develop their careers is particularly pertinent to agriculture, where there are worldwide workforce shortages and misconceptions about the nature of agricultural work (Christiaensen et al., 2020; Ryan, 2023). Worldwide research shows that many young people lack agricultural understanding (Beecher et al., 2019; Hess and Trexler, 2011; LEAF, 2023; Manning et al., 2024; Smeds et al., 2015). Often those who influence and support young people in person (Beecher et al., 2019; Cosby et al., 2024; McDonald et al., 2025) are similarly limited in their awareness and understanding, and the potential of online influence and information for agricultural careers is under-developed (Unay-Gailhard and Brennen, 2022). When thinking of potential careers in agriculture, young people typically only consider traditional and conventional farming, lacking understanding of modern technology and opportunity, and links to environmental sustainability, and thus may deem the sector unattractive (Consentino et al., 2023; Manning et al., 2024). Yet, when young people are well-informed about work in agriculture it can be perceived as a place of vocational “passion, pride, and purpose” (De Guzman et al., 2025, p.88), offering fulfilment through decent work in line with United Nations Sustainable Development Goal (UN SDG) 8 (United Nations, 2025). Workforce sustainability and resilience is critical to future agricultural success and sustainability (Rose et al., 2021); limited understanding amongst the future workforce paired with existing staffing challenges creates a concerning picture for the agricultural sector and the wider world. What is currently less clear is why, given the known issues around ageing workforces and lack of new entrants to agriculture (Consentino et al., 2023), the situation is persisting rather than resolving (Ryan, 2023). This paper uses qualitative content analysis (QCA) to analyse the extent of any consensus amongst varied UK stakeholders on the question of how to increase agricultural understanding and awareness amongst young people, and whether they approach the current situation with criticality and/or constructiveness. Understanding current stakeholder perspectives and willingness to act has the potential to support understanding of why significant sector challenges persist, and how they might be overcome.

The analysis uses a dataset of response to the United Kingdom (UK) Parliament's Environment, Food and Rural Affairs (EFRA) Committee, and their inquiry into Education and Careers in Land-based Sectors (Environment, Food and Rural Affairs Committee, 2023) which invited written responses from stakeholders from September to November 2023. exploring land-based sector awareness and career development opportunities in the UK. This paper focuses on how a range of agricultural sector stakeholders responded to question one (Q1) in the inquiry “How can the understanding and awareness of career opportunities in land-based sectors be improved among children and young adults?” (Environment, Food and Rural Affairs Committee, 2023), exploring the extent of stakeholder consensus, constructiveness and criticism. The overall inquiry extended to eight questions, covering a range of topics related to agricultural careers including a focus on education. This analysis focuses solely on Q1 as, in line with SCCT, building understanding and awareness is likely to be a key starting point for any subsequent action or policy change in agricultural education and/or careers.

## 2 Method

### 2.1 Qualitative content analysis (QCA)

Qualitative content analysis (QCA) has been used in other studies and research based on responses to government inquiries (Condie et al., 2022; Wiley and Goulding, 2023). In this research it was used to group and analyse the written inquiry Q1 submissions, to establish whether there is consensus amongst varied stakeholders on how to increase agriculture awareness amongst children and young people, including consideration of stakeholder constructiveness and criticism, through analysis and synthesis of the inquiry submissions. The method draws on that outlined by Condie et al. (2022) in their analysis of attitudes to aquaculture demonstrated in submissions to inquiries by the Australian Senate and the Tasmanian Legislative Council, where QCA was used to group stakeholders, determine submission tone, identify issues, and stakeholder attitudes to those issues. Their successful use of QCA suggests that it is appropriate in this context, given potential parallels in stakeholders across aquaculture and agriculture, and similarities in UK and Australian policy and governance.

QCA supports sense-making through pattern identification and recognition in written or spoken text, with consideration of implicit and explicit content, themes and meaning (Bengtsson, 2016; Lyhne et al., 2025; Preiser et al., 2022; Sirilakshmi et al., 2024). As Condie et al. (2022, p. 2) note, when QCA is used in the context of formal submission data it centres the stakeholders' issues and concerns, and their attitudes towards those. The QCA process used is outlined below.

### 2.2 QCA process

**Figure 1:**
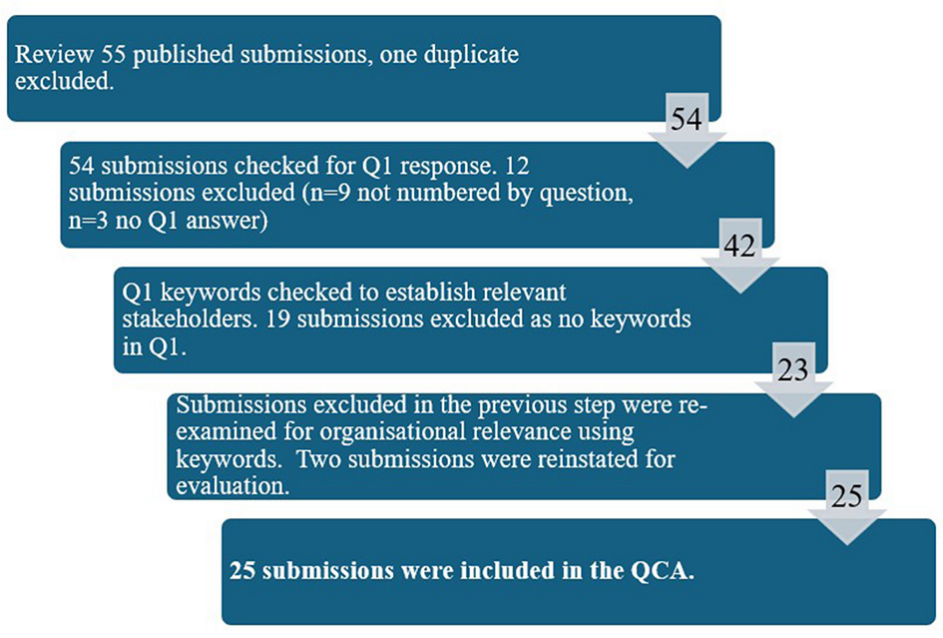
Submission review process.

**Figure 2:**
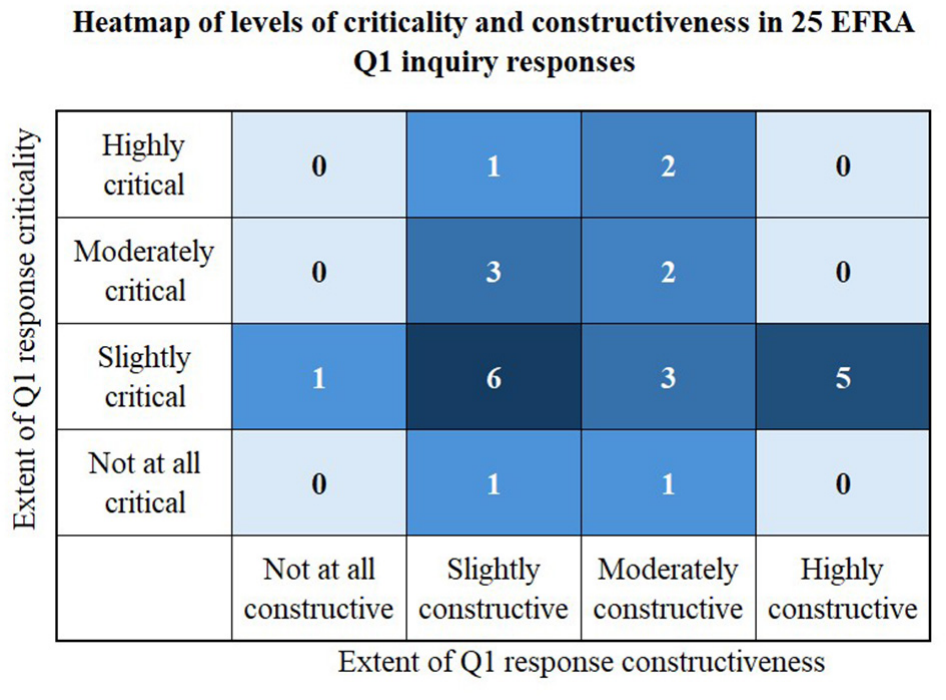
Heatmap of levels of criticality and constructiveness in inquiry responses.

The process and outputs from Sections 2.2.1 and 2.2.2 are shown in Figure 1, results from Section 2.2.3 are shown in Table 1, and results from Section 2.2.4 are shown in Figure 2. Finally, the results from Section 2.2.5 are explored in Sections 3.1–3.4. Where submissions are quoted their inquiry submission number (Environment, Food and Rural Affairs Committee, 2023) is used as the reference.

**Table 1:** Number of submissions received from relevant stakeholders, by stakeholder group.

**Stakeholder groups**	**Number of submissions from stakeholder group**
Regional body	6
Educational body or institution	6
Land-based sector body	5
Individual	3
Commercial organisation	2
Agricultural sector body	2
Careers sector body	1

#### 2.2.1 Check responses

Published written submissions (Environment, Food and Rural Affairs Committee, 2023) were checked for any publication errors; 55 submissions were published, one duplicate submission was excluded from this analysis.

#### 2.2.2 Identify relevant stakeholder submissions

##### 2.2.2.1 Check for clear answer to Q1

The researcher checked all submissions. Where Q1 had not been answered, or the submission was unclear on which inquiry questions were being responded to, it was excluded.

##### 2.2.2.2 Check Q1 response text

Q1 response text was then checked. The submitter was deemed a relevant stakeholder if the response included any of the following keywords: farm, farmer, farming, agriculture, agricultural, or agri- as a prefix. This supported the inclusion of submissions with a perspective on agricultural careers as part of a wider land-based remit, while excluding those solely focused on another area of land-based work.

##### 2.2.2.3 Evaluate organisational/individual description within submission

For those excluded at step 2b, a further check took place, examining the organisational or individual description provided within their submission. If this included any of the keywords above, they were reinstated as a relevant stakeholder. This step was added as some submissions outlined their particular area of interest in relation to their organisation and then used wording such as “this area” or “the sector” in their Q1 response.

These steps produced 25 inquiry responses for inclusion in this analysis. The full responses varied from 1 to 16 pages, with a mean and median of 6 pages. Responses to Q1 varied in length from 76 to 1,106 words, with a mean of 349 words, and a median of 286 words. These results are in line with inquiry guidance around brevity and conciseness (Environment, Food and Rural Affairs Committee, 2024), while providing enough detail and rich content for valid and reliable QCA.

#### 2.2.3 Identify stakeholder groups

As in Condie et al. (2022), relevant stakeholders were then inductively grouped, based on their own description of themselves or their organisation. Where necessary, this was supplemented with a web search to categorise the stakeholder correctly.

#### 2.2.4 Assess submission tone

The Q1 response was read multiple times, to allow particular points of relevance to the research question to be drawn out, alongside careful, objective consideration of the nature and tone of the entire response. The approach taken was hermeneutic in nature i.e., recognising the dialectic relationship between each part of the response, and the response as a whole (Lyhne et al., 2025). The overall response tone was categorised in two ways.

- To what extent was the tone critical in relation to the current situation? (Scale—Not at all critical/Slightly critical/Moderately critical/Highly critical) Responses were situated on this scale based on the extent to which they critiqued or challenged current policy and practise, as a proportion of the whole response i.e., responses that made one or more critical comments in relation to the majority of topics they raised were assessed as “highly critical”, where responses that made critical comments in relation to around half of the topics they raised, or several critical comments on one key topic were “moderately critical”. Where critical comments were present in relation to less than half of the topics raised, responses were categorised as “slightly critical” and no critical comments meant the response was assessed as “not at all critical”. Examples of comments deemed critical include “The opportunity to use agriculture and horticulture for contextual learning across the curriculum is large, but rarely taken advantage of, meaning children miss vital touch points.” (ECL0037) and “There is a long way to go to reverse the erosion of the role of lead bodies in supporting a careers information and awareness campaign.” (ECL0010).
- To what extent was the tone constructive in nature? (Scale—Not at all constructive/Slightly constructive/Moderately constructive/Highly constructive) As above, responses were assessed based on the proportion of topics with constructive comments, ideas or challenges, when the response was looked at in its entirety e.g. “a focus on increasing awareness of land-based careers amongst careers advisers and teachers is as important as a focus on awareness amongst learners themselves. This could also be supported by increased employer interventions in schools.” (ECL0024).

This systematic and structured approach was used to enhance reliability and validity (Sirilakshmi et al., 2024). Exploring constructiveness through appropriate suggested action and supportive engagement with issues, alongside the extent to which each submission was critical, develops understanding of how stakeholders currently feel about the situation, alongside what they feel should be done, supporting understanding through QCA (Preiser et al., 2022). This approach recognises that inquiry submissions can include both negative and positive sentiments, and can be critical whilst also sharing constructive ideas and challenges.

#### 2.2.5 Identify issues raised, and attitudes or feelings related to these issues

##### 2.2.5.1 Initial inductive coding

All Q1 responses were re-read and inductively coded, leading to the identification of 12 initial sub-themes.

##### 2.2.5.2 Coding reliability review

Responses were rigorously reviewed against the complete sub-theme list to support coding reliability, correcting any errors or oversights (Bengtsson, 2016). It is noted that this work was completed by a sole researcher; validity could be increased in future work by cross-checking coding with another researcher or software designed for this purpose.

##### 2.2.5.3 Thematic group creation

The 12 identified sub-themes were then grouped into four thematic groups, each comprising two to four sub-themes.

##### 2.2.5.4 Attitudes/feelings identified

Responses were read again and common attitudes or feelings expressed in relation to each thematic group and sub-theme were identified.

### 2.3 QCA reliability and validity

In QCA, reliability and validity relate to the generation of findings that provide insight into the matter being studied (Lyhne et al., 2025; Preiser et al., 2022). All possible steps should be taken to support internal reliability and validity and reduce bias in the research process (Condie et al., 2022; Noble and Smith, 2015), including any bias or influence from the researcher themselves (Bengtsson, 2016). In this work, this included an objective and content-neutral approach to stakeholder inclusion, which took place before content review. The researcher also used a self-critical and iterative review process e.g. adding the step described in Section 2.2.2.3 after reflecting on how submissions had been worded, and the iterative approach in Section 2.2.5, where multiple reviews of the same data supported consistent coding and opportunities for researcher self-reflexivity.

## 3 Results

Using the QCA process outlined in Section 2.2, 25 submissions were further categorised and evaluated for tone and issues raised.

Table 1 shows that the 25 submissions included in this evaluation come from a range of relevant stakeholders with an interest in children and young adults' understanding and awareness of agricultural careers. As expected, the majority were directly linked with agriculture or the wider land-based sector, for example, many of the educational bodies or institutions delivered primarily, or solely, land-based courses and qualifications. Where sector bodies were agricultural in focus they were categorised as agricultural sector bodies. Where they focused on agriculture alongside other areas of land-based activity they were categorised as a land-based sector body. There were no obvious crucial stakeholder groups who were unrepresented in the responses.

Figure 2 highlights that the majority of responses were slightly critical, and either slightly, moderately or highly constructive. The most constructive responses typically came from agricultural sector bodies, followed by educational bodies or institutions, and regional bodies. The most critical responses came from regional bodies, followed by agricultural sector bodies and land-based sector bodies. From these highly engaged stakeholder groups it was common for responses to be both constructive and critical, recognising and engaging with complex issues, challenging current practise and supportive suggesting appropriate ways forward. Individual respondents were the least constructive group, often having a more limited perspective on some of the issues and perhaps less agency to create change. Overall responses were typically more constructive than critical, demonstrating positive engagement with the question of how to enhance children and young adults' understanding and awareness of agricultural careers.

The issues raised in each submission, and the attitudes or feelings related to these issues are explained below, using the four thematic groups to consider the 12 sub-themes.

### 3.1 Thematic group one—high quality career and labour market information and support

#### 3.1.1 Thematic group one—overview and sub-themes

A significant majority of stakeholders (*n* = 20) identified a need to develop careers education, information, advice and guidance (CEIAG) and agricultural labour market information (*Sub-theme 1*), as noted here, “Schools and career services need to be able to access curated, transparent advice on career pathways and educational opportunities, as should parents, guardians and students” (ECL0010). Many felt that this should include sector-specific support or training for those delivering CEIAG, and centralised specialist resources. Five stakeholders specifically mentioned the parents or guardians of young people in their Q1 response, highlighting their influence, and the need for them to be better informed to prevent misinformation (*Sub-theme 2*). Seventeen stakeholders wrote more broadly about the need to dispel myths e.g. agriculture is a low pay/low skill sector, and to increase young people's understanding of modern agricultural opportunities and pathways (*Sub-theme 3*). The final sub-theme in this category was the importance of supporting and/or delivering research into sector careers to inform practise (*Sub-theme 4*). Eight stakeholders added their thoughts on this, with many highlighting existing projects and activities in this space. The following response captures many of these themes

“There is still the need to dispel the misconception around the types of careers and opportunities available, including salary expectations, and that these are not restricted to the more conventional expectations of traditional farming and agricultural roles. It should show a vibrant and progressive industry with opportunities for many different skills and roles, together with the benefits. This should not be just focused on children and young adults, but amongst teachers, careers advisors, family/carers and JobCentre Plus staff.” (ECL0034)

#### 3.1.2 Thematic group one—agreement between stakeholders

Respondents from all stakeholder groups identified two or more sub-themes within this thematic group in their submission; there was unanimous consensus on its importance.

#### 3.1.3 Thematic group one—attitudes of stakeholders

Typically, submissions were positive and constructive about the impact that high quality CEIAG and labour market information could have on children and young adult's understanding and awareness of agricultural careers. However, more negative or critical comments were common in relation to the myths or outdated perceptions of agricultural sector employment, and poor advice and choices that might stem from these, with respondents feeling that “The sector needs to increase the visibility of land-based career options, but also improve the information that is shared, to ensure it is aspirational, accurate and looks towards the highly skilled, technical, STEM careers that exist and are emerging in the sector.” (ECL0023).

### 3.2 Thematic group two—engaging with education

#### 3.2.1 Thematic group two—overview and sub-themes

This thematic group focused specifically on educational settings, considering activities in curriculum and beyond, and how teachers and educators might play a key role in agricultural awareness raising. Over three quarters of stakeholders (*n* = 19) noted the need for the delivery of in-curriculum educational activity and any associated support for educators to support their own understanding (Sub-theme 5). Some submissions highlighted the need for agriculture to be clearly aligned to the STE(A)M subject grouping (Science, Technology, Engineering, (Arts) and Maths) and for suitable qualifications to be available. The need for a sustained focus on agricultural learning in secondary schools was also commented upon in these responses, as illustrated below:

“…it is surprising that land-based industries such as agriculture, horticulture and related subjects are rarely perceived as a career choice in schools during key stage 3–4+. There is great scope for these subjects to be introduced across the national […] STEAM curriculum […]. Primary schools are typically very good at introducing young people to plants and nature with strong links to food, the environment and sustainability, though this is more difficult in urban areas. However, this narrative seems to be lost when young people graduate to secondary school.” (ECL0044)

Ten stakeholders also considered the potential for co-curricular or extra-curricular activities to build further agricultural sector awareness (Sub-theme 6), with suggestions like “Incorporate agroecological farming, food production and ecological forestry into the school curriculum. In addition to the formal curriculum itself, this can include onsite school growing projects as well as farm visits.” (ECL0046).

#### 3.2.2 Thematic group two —agreement between stakeholders

Respondents from all stakeholder groups (apart from the careers sector body) identified both of the sub-themes within this thematic group in their submissions. There was therefore a high level of general consensus across the majority of stakeholders as to the importance of education and educators.

#### 3.2.3 Thematic group two—attitudes of stakeholders

Where comments were critical in this thematic group, this sentiment often related to wider UK policy that had limited the time and resource in educational settings to deliver agriculturally-focused activities, with an impact on children and young adults' understanding and awareness of agricultural careers. Some stakeholders felt teachers were not always knowledgeable in relation to agriculture, for example, “Feedback from providers of outdoor learning services to schools makes it clear that teachers are less connected and less confident and competent in natural environment settings than they were a generation ago.” (ECL0017) but most also made constructive suggestions relating to training, activities or support.

### 3.3 Thematic group three—collaboration, partnership and policy

#### 3.3.1 Thematic group three—overview and sub-themes

Seventeen stakeholders highlighted the vital importance of collaboration and partnership working across sector bodies, national and regional bodies, government, educators and employers etc. (*Sub-theme 7*). This was shared either with examples of their existing activities and/or through calls for more collaborative activity, such one respondent who shared “…we produced a report recommending next steps to raise awareness of sustainability in the agri-food industry for young-people. These recommendations include offering experiential learning opportunities to allow young people to experience and learn about sustainable agriculture first hand, including experiencing the supply chain.” (ECL0030). This sub-theme also included calls for employer engagement, as a form of collaboration, and within that, that employers support and develop enhanced opportunities for young people to engage in work-based learning. Finally, within this thematic group, eight stakeholders called for better funding and financial support for agricultural training and careers, as part of regional and national policy (*Sub-theme 8*). Some respondents alluded specifically to regional and national need for better funding of CEIAG activity in education and beyond.

#### 3.3.2 Thematic group three—agreement between stakeholders

Respondents from all stakeholder groups contributed to at least one sub-theme within this thematic group, indicating unanimous consensus on its overall importance.

#### 3.3.3 Thematic group three—attitudes of stakeholders

There was an overwhelmingly positive and constructive attitude to the potential for improved collaboration and partnership working, with examples such as the regional one below highlighted in responses.

“In Lancashire, the Lancashire Skills and Employment Hub, using DfE Skills Advisory Panel funds, undertook a deep dive into the local Food and Agriculture sector, working collaboratively with the Lancashire Enterprise Partnership Sector Panel (including the NFU, local businesses (large and small) and Lancashire's land-based college, Myerscough). This study […] has underpinned the activity of the Lancashire Careers Hub to enable young people and adults understand the job market.” (ECL0042)

Where more critical comments arose these related to the policies or financial challenges that made such activities more challenging to deliver, and potentially undermined efforts to increase children and young adult's understanding and awareness of agricultural careers. One respondent called on the inquiry to “Improve funding to land based providers so we can support by providing experts to schools and supporting school initiatives where they are looking at important national/global priorities around environment and food sustainability for example.” (ECL0045).

### 3.4 Thematic group four—public presence and awareness

#### 3.4.1 Thematic group four—overview and sub-themes

The final thematic group focused on public engagement and awareness, as a conduit to children and young people, as shown in the quote below.

“A communication campaign: to change public perception and to demonstrate that agriculture is vital to the economy and food security; highlighting that there is a vast array of jobs at all levels appealing to students with practical and more academic skills; and stressing that jobs in agriculture promote and improve not only food production but also environmental sustainability, levelling up and net zero ambitions.” (ECL0031)

A key aspect of this was the need for agriculture to be clearly and accurately linked to matters of wider environmental sustainability, climate change and the food chain, which were perceived to be public interest (*Sub-theme 9*). This was raised by over two-thirds (*n* = 17) of the stakeholders in their submissions. Twelve stakeholders also suggested that those within the agricultural sector should develop their online presence and/or support public engagement (*Sub-theme 10*), with two stakeholders specifically alluding to the importance of role models and case studies (*Sub-theme 11*) for example, “Case studies […] showing aspirational farmers as role models, the high-tech industry, and the care for animal welfare and the environment to break with any misplaced old stereotypes will help.” (ECL0041). Four submissions made clear mention of diversity and/or inclusion in awareness raising (*Sub-theme 12*), including this allusion to a specific project “How Farming Can Cool the Planet […] demonstrates how young people have come together to understand how farming can play a part in tackling the climate emergency. Funded by Farming the Future, this is a collaborative project […] and involves young people from a diverse range of backgrounds.” (ECL0007). This may be because other stakeholders presumed this to be self-evident, or tackled this in a separate element of their inquiry response, but it is worthy of note to ensure that this critical aspect of sector engagement is not overlooked.

#### 3.4.2 Thematic group four—agreement between stakeholders

Respondents from all stakeholder groups identified at least two sub-themes from this thematic group in their submissions; there was unanimous consensus on its importance.

#### 3.4.3 Thematic group four—attitudes of stakeholders

Responses and suggestions related to this thematic group were generally constructive and enthusiastic, keen to work collaborative with young people themselves to change perceptions of agriculture and help them develop their understanding and awareness of agricultural careers.

## 4 Discussion

This research aimed to understand the extent of any consensus amongst agricultural stakeholders on the best way to improve the current situation of limited agriculture awareness amongst children and young people. Given stakeholders near unanimous agreement across all thematic groups, and the numerous areas of consensus and common ground within sub-themes, it can be concluded that there is agreement across this group on how to move forward. However, it is worth noting that the analysed submissions came from an engaged group i.e., those invested enough to respond to a government inquiry, and thus may not be fully representative of the wider agricultural sector. Although respondents represented all expected stakeholders, further work could add additional perspectives and insights from a larger, potentially more diverse, group. This should include those who are not currently engaged with agriculture in any way. While UK focused, the findings and recommendations emerging from this research could be of interest in other nations due to worldwide concern about the sustainability of the agricultural workforce. The UN SDGs aim to ensure decent work (SDG 8) for all while moving towards zero hunger (SDG 2), agriculture has a key role to play here (United Nations, 2025).

The development of four thematic groups within this research supported the analysis and synthesis of the suggested actions and activities. Existing research which paints agriculture as a misunderstood sector (Beecher et al., 2019; Christiaensen et al., 2020; Hess and Trexler, 2011; LEAF, 2023; Manning et al., 2024; McDonald et al., 2025; Ryan, 2023) was reinforced by stakeholder comments, and constructive solutions around the introduction of centralised career information, advice and resources that dispel myths and inform children, young adults, and their supporters and influencers were encouraged by stakeholders. Inquiry responses demonstrated that the responding stakeholders had a strong grasp on the contextual factors, influences and environments that typically shape a young person's career development, as outlined in SCCT. Indeed, stakeholders suggested that all those who advise and support young people should be supported to understand modern agricultural careers, and the potential they offer; again this is in line with sentiments that have emerged from earlier research about the attractiveness, or lack thereof, of agricultural careers (Consentino et al., 2023; Manning et al., 2024). This also makes sense given the importance of a young person's wider context, support and environmental influences on their career development and decision-making (Hartung et al., 2005; Howard and Ferrari, 2022; Lent et al., 1994; Sullivan and Baruch, 2009; Watson and McMahon, 2005). To take action in this critical area, the consensus and constructiveness identified across inquiry responses must be harnessed, to develop sector-wide action that improves CEIAG for all young people and those who influence them. As suggested by inquiry respondents, this should include centralised specialist resources for young people, sector-specific support or training for those delivering CEIAG, and associated resources for parents and teachers.

Educational engagement through curriculum that incorporated agriculture as a key element within STEAM, supported by extra-curricular trips and activities, was also considered to be a vital part of developing children and young adult's understanding and awareness of agricultural careers. This included engagement with educators, to support their own development, as they may themselves have limited understanding (Cosby et al., 2024; McDonald et al., 2025). Stakeholders highlighted the need for regional and national policies, and financial support and investment, that facilitate children and young people's awareness of agricultural careers and were often critical of current policy and support, in line with other research calling for greater investment (De Guzman et al., 2025; Ryan, 2023). However, they were highly positive about collaboration and partnership working, both in terms of existing activity, and the potential for future developments. Further enthusiasm was evident around the potential to build broader public awareness of agricultural careers, that supports children and young people, by clearer alignment with environmental sustainability, the management of changing climates, and the food chain, as will be critical in agriculture 4.0 (Rose et al., 2021). Stakeholders also felt that a more coherent online presence would support sector careers, an under-developed area at present (Unay-Gailhard and Brennen, 2022). Finally, the matter of sector diversity and inclusion was touched upon by stakeholders, which is a known challenge (Ryan, 2023), and should be an underpinning consideration in all other areas of action.

The degree of agreement between the existing literature and the stakeholders who responded to the EFRA Committee's call (2023) points to an agricultural sector, and associated stakeholders, who understand the nature and scale of the challenge ahead. The extent of stakeholder consensus and the generally constructive outlook shown in even critical inquiry responses, evidences that this engaged group is keen to tackle this challenge. The actions and activities proposed to increase children and young adults' understanding and awareness of agricultural careers align to wider career development processes and existing careers research. As such, it seems that many of the activities needed to resolve agricultural sector workforce and awareness challenges can be delivered, at least in part, by the relevant stakeholder groups identified in this study. It is recommended that these stakeholders are empowered to develop further collaborations and joint activity, with appropriate government policy and financial support in place, to help them succeed in delivering changes in line with the outcomes of the EFRA Committee's inquiry.

## Data Availability

Publicly available datasets were analysed in this study. This data can be found here: https://committees.parliament.uk/work/7933/education-and-careers-in-landbased-sectors/publications/written-evidence/.
